# A Single Institution’s Experience with Bevacizumab in Combination with Cytotoxic Chemotherapy in Progressive Malignant Glioma

**DOI:** 10.4137/cmo.s827

**Published:** 2008-06-09

**Authors:** Tina Mayer, Jill Lacy, Joachim Baehring

**Affiliations:** Medical Oncology, Yale University School of Medicine, New Haven, CT; Yale Medical Oncology, Yale University School of Medicine, New Haven, CT; Neurology, Yale University School of Medicine, New Haven, CT

**Keywords:** bevacizumab, glioma, glioblastoma multiforme

## Abstract

**Background:**

Bevacizumab and irinotecan may represent one of the most active treatments in progressive malignant glioma. Limited published experience with bevacizumab in patients with CNS tumors raises concerns regarding toxicity, particularly in regards to hemorrhage and thromboembolism.

**Methods:**

We retrospectively reviewed 36 patients with progressive malignant glioma after prior resection, chemotherapy and radiation who were treated with bevacizumab at our institution. Patients were evaluated for bevacizumab-related adverse events, time to treatment failure (TTF) and overall survival (OS). Two patients who progressed or died prior to completion of 4 cycles of therapy were analyzed for adverse events only.

**Results:**

Patients were treated with bevacizumab alone (1), bevacizumab plus irinotecan (31), or bevacizumab plus carboplatin (4). In 34 patients who received >4 cycles of bevacizumab, median TTF and OS were 16 and 32 weeks, respectively. Toxicities included 1 arterial thrombosis, 4 venous thromboses, and 3 clinically significant CNS hemorrhages.

**Conclusion:**

Overall, our results confirm the efficacy and safety of bevacizumab in combination with chemotherapy in patients with progressive malignant glioma. Although the TTF and OS were less than previously reported with the combination of bevacizumab and irinotecan, this was an unselected patient population with 50% of patients having received >1 prior chemotherapy regimen.

## Introduction

Malignant glioma is a devastating disease with median survival of approximately one year for grade IV glioblastoma multiforme (GBM) and three years for grade III anaplastic astrocytoma (AA). The prognosis of patients with recurrent or progressive malignant glioma is extremely poor. Repeat surgery or radiotherapy is often precluded by the morbidity of these interventions, and chemotherapy has limited efficacy. The median survival of patients with recurrent GBM or AA who are enrolled in phase II chemotherapy trials is just 30 weeks and survival is likely lower in patients who are ineligible for trials at the time of their recurrence (Wong et al. 1999). Given the lack of effective therapeutic options, there is no universally accepted standard of care for recurrent malignant glioma, and patients are encouraged to participate in clinical trials evaluating new agents.

Prior to 2005, the standard of care for the primary treatment of malignant gliomas was surgical resection followed by radiotherapy. The benefit of nitrosourea-based chemotherapy was unproven in randomized prospective trials, although a meta-analysis from 12 randomized trials showed an increase in one year survival from 40% to 46% with chemotherapy (Stewart, 2002). The standard of care for newly diagnosed GBM was modified in 2005 to include the non-classical alkylating agent, temozolomide, as adjuvant and concomitant therapy with external beam radiotherapy. This treatment protocol increases median survival from 12.1 months with radiotherapy alone to 14.6 months with the combination of radiotherapy and temozolomide (Stupp et al. 2005). Despite the overall survival benefit from the addition of temozolomide to radiotherapy, 90% of patients developed progressive disease by 24 months.

In view of the poor prognosis of glioma patients despite treatment with conventional chemotherapy, interest has turned towards the combination of targeted therapies with chemotherapy. Malignant gliomas are highly vascularized and thus angiogenesis inhibitors are being actively explored in these tumors. VEGF has been found to be important in the development of abnormal vasculature seen in malignant gliomas (Plate et al. 1992; Chaudhry et al. 2001). Tumors with increased vascular proliferation tend to be more aggressive and the extent of vascularization corresponds with prognosis (Leon et al. 1996; Birner et al. 2003). Bevacizumab is a monoclonal antibody to VEGF, which prevents interaction with VEGF receptors on the cell surface. Bevacizumab has been approved for use in combination with chemotherapy in metastatic colon, breast, and lung cancer (Miller et al. 2007; Giantonio et al. 2006; Ramalingam et al. 2008; Sandler et al. 2006). In malignant gliomas, bevacizumab (10 mg/kg) has been evaluated in combination with irinotecan (125mg/m^2^, or 340mg/m^2^ in patients on EIAEDs, enzyme inducing anti-epileptic drugs) with cycles given every two weeks (Vredenburgh et al. 2007a; Vredenburgh et al. 2007b). Early clinical experience with this regimen in GBM has been promising, with a phase II study of thirty-five recurrent GBM patients showing a median progression-free survival of 24 weeks and median overall survival of 42 weeks (Vredenburgh, 2007b). Thrombotic events were noted, though risk of CNS hemorrhage was minimal. These results have demonstrated the efficacy and safety of bevacizumab in combination with irinotecan in a small number of patients with malignant glioma. Bevacizumab seems to be well tolerated and response rates are amongst the highest ever reported in this population. Validation in a larger study is pending and toxicity data are limited. Over the past 24 months, we have used bevacizumab-based salvage chemotherapy in patients with recurrent or progressive gliomas at our institution and report our experience herein.

## Methods

We performed a retrospective analysis of patients treated at our institution between December 2005 and December 2007 with bevacizumab-based salvage therapy, after prior standard therapy for progressive malignant glioma. Patients had to have histopathologic evidence of an infiltrative glioma and radiographic evidence of malignant tumor progression (enlarging area of nodular contrast enhancement). Failure of standard of care therapy (radiation, temozolomide) had to be documented. Patients eligible for a clinical trial available at our institution were treated with bevacizumab-based salvage therapy only if they were not interested in participating in the trial. Patients were evaluated for bevacizumab-related adverse events, time to treatment failure (TTF), and overall survival (OS). Overall survival was determined based on first date of treatment with this regimen until date of death from any cause. In two patients, the exact date of death was unknown and the date of last correspondence was used. TTF was determined based on first date of treatment with this regimen until progression, death, unacceptable toxicity or patient decision to withdraw from treatment. Median times and confidence intervals were calculated by SigmaPlot^®^ statistics program. Two patients who progressed or died prior to completion of four cycles of bevacizumab were analyzed for adverse events only. The bevacizumab dose ranged from 5–10 mg/kg, typically given every two weeks. The second agent was irinotecan at 100–200mg/m^2^ (up to 340mg/m^2^ on patients on EIAEDs) or carboplatin with AUC of 5–6. Hemorrhage that led to new symptoms, decline in status, or hospitalization were considered clinically significant (as per CTCAE 3.0 grading of adverse events, 2006) as opposed to hemorrhage found incidentally on restaging studies.

## Results

In thirty-six patients treated with bevacizumab-based regimens, the median age was 50 (range 24–76). Twenty-two patients had progressive GBM and fourteen patients had other progressive gliomas, including: grade III oligodendroglioma (4), grade III astrocytoma (4), grade III ependyoma (1), grade III mixed glioma (1) and grade II glioma with clear evidence of progression (4). In four patients, progression from grade II to grade III or IV was presumed based on imaging and clinical status even though a biopsy was not repeated at the time of progression. The other thirty-two had biopsy proven malignant glioma. All patients had received prior radiotherapy and temozolomide. Eighteen patients (50%) had received temozolomide only, and eighteen (50%) had received temozolomide and other salvage treatments as follows: AZD2171 (cediranib), ST1481 (gimatecan), erlotinib/rapamycin, imatinib/hydroxyurea, thalidomide/temozolomide/celebrex, IL-13 pseudomonas exotoxin, PCV, and high dose carboplatin/etoposide with autologous stem cell transplant. Twenty-two percent of patients had already undergone at least three prior chemotherapies.

**Figure 1:**
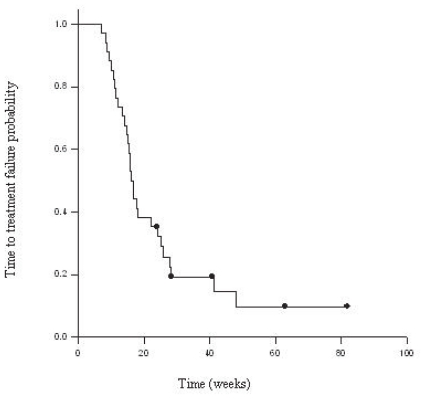
Kaplan-Meier for time to treatment failure.

**Figure 2:**
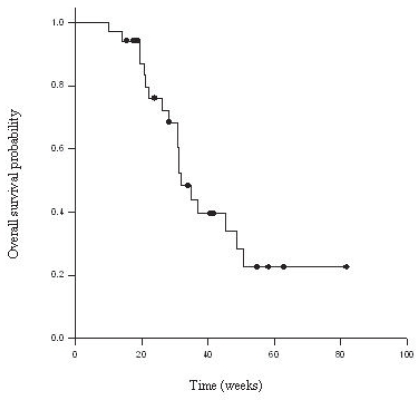
Kaplan-Meier for overall survival.

Thirty-six patients were treated with bevacizumab-based regimens as follows: bevacizumab alone (1), bevacizumab and irinotecan (31), and bevacizumab and carboplatin (4). Thirty-four received ≥4 cycles of bevacizumab (median 8, range of 4–23 cycles), and were included in efficacy analysis. The median TTF was 16 weeks [14.2–17.8, 95% C.I.] with a range of 7–82 weeks (See Fig. 1). The median OS was 32 weeks [27.2–36.6, 95% C.I.] with a range of 10–82 weeks (See Fig. 2). The progression free survival at 6 months was 25%. Six patients were still being actively treated at the time of this analysis.

Review of the adverse events in the thirty-six patients revealed one arterial thrombotic event, specifically myocardial infarction (2.8%), four venous thrombotic events (11.1%), and five intracranial hemorrhages, of which only three were clinically significant (8.3%). There was also one non-clinically significant intracranial hemorrhage one month after the last treatment.

## Discussion

Many of our patients were previously on clinical trials and were not eligible for additional trials at the time of their bevacizumab therapy. Their treatment options were extremely limited. A meta-analysis of eight phase II chemotherapy trials in 375 patients with recurrent GBM and AA after radiotherapy found an overall progression free survival at 6 months of 21% (15% for GBM, 20% for AA), median progression free survival of 10 weeks and median overall survival of 30 weeks (Wong, 1999). In this analysis, one trial excluded patients with any previous chemotherapy and two trials excluded patients with more than one previous chemotherapy regimen. The overall survival in our patients was similar to that reported in patients being enrolled in chemotherapy clinical trials.

The two published phase II studies with bevacizumab in combination with irinotecan have raised the possibility that this combination may represent the most active regimen in progressive malignant gliomas (Vredenburgh et al. 2007a; Vredenburgh et al. 2007b). The first of these studies included 32 patients with recurrent malignant gliomas (23 grade IV, 9 grade III lesions) (Vredenburgh et al. 2007a). The median PFS was 23 weeks for all (20 weeks for grade IV and 30 for grade III). Toxicities in this group of patients included three venous thrombotic events, one arterial ischemic stroke and no CNS hemorrhages. The results for patients with GBM only were reported separately with an additional 12 patients, demonstrating PFS of 24 weeks and median survival of 42 weeks in this subset of 35 patients (Vredenburgh et al. 2007b). In this group, four venous thrombotic events and one CNS hemorrhage were observed.

The median TTF of 16 weeks in our series was lower than that of 23–24 weeks reported in the published phase II studies with irinotecan and bevacizumab. This may reflect patient selection, as 50% of the patients in our series had received prior salvage therapy after failing temozolomide, including eight (22%) patients who had received three or more prior regimens. Our results were similar to or better than trials with other experimental targeted therapies in patients with progressive gliomas. One of the more promising of these agents is cediranib (AZD2171), an oral VEGF and PDGF receptor TK inhibitor which has been shown to normalize vasculature in patients with recurrent glioblastoma (Batchelor et al. 2007a). In a phase II trial of AZD2171 in recurrent GBM, the 6 month PFS was 27.6%, median PFS was 16 weeks, and median overall survival was 32 weeks (Batchelor et al. 2007b).

Numerous other agents are being tested including vatalanib, vorinostat, lenalidomide, erlotinib, and enzastaruin and preliminary data shows limited activity (Fine et al. 2007; Galanis et al. 2007; Cloughesy et al. 2005; Conrad et al. 2004; Reardon et al. 2004; Fine et al. 2005). Published phase II data is available on imatinib, thalidomide, gefitinib and temsirolimus. Treatment with imatinib mesylate, which is an inhibitor of multiple tyrosine kinase inhibitors including PDGFR, in combination with hydroxyurea, was found have a median progression-free survival (PFS) of 14.4 weeks and PFS at 6 months of 27% in patients with recurrent GBM (Reardon et al. 2005). Thalidomide has also been evaluated for its anti-angiogenic properties. As a single agent, thalidomide was found to have a median survival of 31 weeks and 6 month PFS of 18% in patients with recurrent glioblastoma (Marx et al. 2001). In patients with recurrent high grade gliomas, the median TTP was 10 weeks with a median overall survival of 28 weeks (Fine et al. 2000). In combination with carmustine, thalidomide was found to have 6 month PFS of 27% and median PFS of 14 weeks (Fine et al. 2003). Single agent gefitinib produced a median TTP of 8.4 weeks and median OS of 24.6 weeks, with a 14% PFS at 6 months (Franceschi et al. 2007). Temsirolimus was not found to have significant efficacy, with a median time to progression of 9–10 weeks in patients with recurrent GBM (Galanis et al. 2005; Chang et al. 2005). Of all these studies, none of the agents showed efficacy as impressive as that published with irinotecan and bevacizumab. However, there is considerable concern about the potential toxicities of using bevacizumab in the glioma population.

Bevacizumab causes mild mucosal bleeding and has been associated with life-threatening pulmonary hemorrhage in a subset of patients with lung cancer, raising concerns about risk of intra-cranial hemorrhage in patients with gliomas (Sandler et al. 2006). Studies with bevacizumab in patients with various types of cancer have excluded patients with brain metastases given concern over risk of CNS bleeds (Sandler et al. 2006; Hurwitz et al. 2005). Our study found clinically significant hemorrhages in three patients (8%). Two of these hemorrhages were in patients on anticoagulation with either low molecular weight heparin or warfarin. Our patients clearly had more toxicity in terms of hemorrhage compared with previously reported data with bevacizumab, but 8 (22%) of our patients were on anticoagulation whereas the previously published study excluded patients who were on anticoagulation (Vredenburgh et al. 2007a). In addition, those that developed need for anticoagulation during treatment with bevacizumab were taken off study (Vredenburgh et al. 2007a). Patients with baseline hemorrhage on MRI were excluded in the previous study, though it is not clear if patients with history of prior hemorrhage were excluded. One of the patients treated at our institution who developed CNS hemorrhage had a history of hemorrhage at presentation two years prior. Glioblastomas are known to have a significant risk of hemorrhage. The incidence of spontaneous intracranial hemorrhage in patients with gliomas ranges from 6.2%–8.7% (Wakai et al. 1982; Kondziolka et al. 1987; Lieu et al. 1999). In addition to hemorrhage, concerns have also been raised about craniotomy site dehiscence in patients treated with bevacizumab (Chamberlain, 2008). In our review, two patients were noted to have delayed wound healing after port placement but otherwise no wound issues were noted.

Overall, our results have demonstrated clinical efficacy of bevacizumab-based regimens in patients with progressive malignant gliomas. This was an unselected patient population with 50% of patients having received more than one prior regimen, some with previous antiangiogenic agents. Thus it is not surprising that our TTF and OS are less than that seen in the two published studies with bevacizumab and irinotecan. We observed no unusual or excessive toxicities of bevacizumab in these patients. Finally, our experience supports the prior observation that bevacizumab does not substantially increase the risk of intracranial bleed above the baseline risk of bleeds in these tumors, though one should use caution in patients requiring anticoagulation.

## References

[1] Wong ET, Hes KR, Gleason MJ (1999). Outcomes and prognostic factors in recurrent glioma patients enrolled onto phase II clinical trials. J Clin Oncol.

[2] Stewart LA (2002). Chemotherapy in adult high-grade glioma: a systematic review and meta-analysis of individual patient data from 12 randomized trials. Lancet.

[3] Stupp R, Mason WP, van den Bent MJ (2005). Radiotherapy plus concomitant and adjuvant temozolomide for glioblastoma. New Engl J Med.

[4] Plate KH, Breier G, Weich HA (1992). Vascular endothelial growth factor is a potential tumour angiogenesis factor in human gliomas in vivo. Nature.

[5] Chaudhry IH, O’Donovan DG, Brenchley PE (2001). Vascular endothelial growth factor expression correlates with tumour grade and vascularity in gliomas. Histopathology.

[6] Leon SP, Folkerth RD, Black PM (1996). Microvessel density is a prognostic indicator for patients with astroglial tumors. Cancer.

[7] Birner P, Piribauer M, Fischer I (2003). Vascular patterns in glioblastoma influence clinical outcome and associate with variable expression of angiogenic proteins: evidence for distinct angiogenic subtypes. Brain Pathol.

[8] Miller K, Wang M, Gralow J (2007). Paclitaxel plus bevacizumab versus paclitaxel alone for metastatic breast cancer. N Engl J Med.

[9] Giantonio BJ, Levy DE, O’dwyer PJ (2006). A phase II study of high-dose bevacizumab in combination with irinotecan, 5-fluorouracil, leucovorin, as initial therapy for advanced colorectal cancer: results from the Eastern Cooperative Oncology Group study E2200. Ann Oncol.

[10] Ramalingam SS, Dahlberg SE, Langer CJ (2008). Outcomes for elderly, advanced-stage non small-cell lung cancer patients treated with bevacizumab in combination with carboplatin and paclitaxel: analysis of eastern cooperative oncology group trial 4599. J Clin Oncol.

[11] Sandler A, Gray R, Perry MC (2006). Paclitaxel-carboplatin alone or with bevacizumab for non-small cell lung cancer. N Engl J Med.

[12] Vredenburgh JJ, Desjardins A, Herndon JE (2007a). Phase II Trial of Bevacizumab and Irinotecan in Recurrent Malignant Glioma. Clin Cancer Res.

[13] Vredenburgh JJ, Desjardins A, Herndon JE (2007b). Bevacizumab Plus Irinotecan in Recurrent Glioblastoma Multiforme. J Clin Oncol.

[14] Common Terminology Criteria for Adverse Events v3.0 (CTCAE) (2006). http://ctep.cancer.gov/forms/CTCAEv3.pdf.

[15] Batchelor TT, Sorensen AG, di Tomaso E (2007a). AZD2171, a pan-VEGF receptor tyrosine kinase inhibitor, normalizes tumor vasculature and alleviates edema in glioblastoma patients. Cancer Cell.

[16] Batchelor T, Sorensen AG, Ancukieicz M (2007b). A phase II trial of AZD2171 (cediranib), an oral pan-VEGF receptor tyrosine kinase inhibitor, in patients with recurrent glioblastoma. J. Clin. Oncol., 2007 ASCO Annual Meeting Proceedings Part I.

[17] Fine HA, Kim L, Albert PS (2007). A phase I trial of lenalidomide in patients with recurrent primary central nervous system tumors. Clin Cancer Res.

[18] Galanis E, Jaeckle KA, Maurer MJ (2007). N.047B.: NCCTG phase II trial of vorinostat (suberoylanilide hydroxamic acid) in recurrent glioblastoma multiforme (GBM). J. Clin. Oncol..

[19] Cloughesy T, Yung A, Vrendenberg J (2005). Phase II study of erlotinib in recurrent GBM: Molecular predictors of outcome. J. Clin. Oncol., 2005 ASCO Annual Meeting Proceedings.

[20] Conrad C, Friedman HS, Reardon DA (2004). A phase I/II trial of single agent PTK787/ZK 222584 (PTK/ZK), a novel, oral angiogenesis inhibitor, in patients with recurrent glioblastoma multiforme (GBM). J. Clin. Oncol., 2004 ASCO Annual Meeting Proceedings (Post-Meeting edition).

[21] Reardon DA, Friedman HS, Yung WK (2004). A phase I/II trial of single agent PTK787/ZK 222584 (PTK/ZK), a novel, oral angiogenesis inhibitor, in combination with either temozolomide or lomustine for patients with recurrent glioblastoma multiforme (GBM) [abstract 1513]. Proc. Am. Soc. Clin. Oncol..

[22] Fine HA, Kim L, Royce C (2005). Results from phase II trial of Enzastaurin (LY.317615) in patients with recurrent high grade gliomas (abstract). J. Clin. Oncol..

[23] Reardon DA, Egorin MJ, Quinn JA (2005). Phase II study of imatinib mesylate plus hydroxyurea in adults with recurrent glioblastoma multiforme. J Clin Oncol.

[24] Marx GM, Pavlakis N, McCowatt S (2001). Phase II study of thalidomide in the treatment of recurrent glioblastoma multiforme. J Neurooncol.

[25] Fine HA, Figg WD, Jaeckle K (2000). Phase II trial of the antiangiogenic agent thalidomide in patients with recurrent high-grade gliomas. J Clin Oncol.

[26] Fine HA, Wen PY, Maher EA (2003). Phase II trial of thalidomide and carmustine for patients with recurrent high-grade gliomas. J Clin Oncol.

[27] Franceschi E, Cavallo G, Lonardi S (2007). Gefitinib in patients with progressive high-grade gliomas: a multicentre phase II study by Gruppo Italiano Cooperativo di Neuro-Oncologia (GICNO). Br J Cancer.

[28] Galanis E, Buckner JC, Maurer MJ (2005). Phase II trial of temsirolimus (CCI-779) in recurrent glioblastoma multiforme: a North Central Cancer Treatment Group Study. J Clin Oncol.

[29] Chang SM, Wen P, Cloughesy T (2005). Phase II study of CCI-779 in patients with recurrent glioblastoma multiforme. Invest New Drugs.

[30] Hurwitz HI, Fehrenbacher L, Hainsworth JD (2005). Bevacizumab in combination with fluorouracil and leucovorin: an active regimen for first-line metastatic colorectal cancer. J Clin Oncol.

[31] Wakai S, Yamakawa K, Manaka S (1982). Spontaneous intracranial hemorrhage caused by brain tumor: its incidence and clinical significance. Neurosurgery.

[32] Kondziolka D, Bernstein M, Resch L (1987). Significance of hemorrhage into brain tumors: clinicopathological study. J Neurosurg.

[33] Lieu AS, Hwang SL, Howng SL (1999). Brain tumors with hemorrhage. J Formos Med Assoc.

[34] Chamberlain MC (2008). Bevacizumab plus irinotecan in recurrent glioblastoma, Letter to Editor. J Clin Oncol.

